# *In vitro* intestinal simulation system on the carbon source utilization characteristics and mechanism of interspecific syntrophic effects of *Bifidobacterium longum* CECT7894 and *Pediococcus pentosaceus* CECT8330

**DOI:** 10.3389/fped.2023.1276846

**Published:** 2023-12-14

**Authors:** Jinjun Li, Lei Xu, Jiahao Liao, Xiaoqiong Li, Xin Wang, Qinbin Wu, Liying Zhu

**Affiliations:** ^1^ State Key Laboratory for Managing Biotic and Chemical Threats to the Quality and Safety of Agro-Products, Zhejiang Academy of Agricultural Sciences, Hangzhou, China; ^2^ Institute of Food Science, Zhejiang Academy of Agricultural Sciences, Hangzhou, China; ^3^ Department of Orthopedics, The First Hospital of Shanxi Medical University, Taiyuan, China; ^4^ College of Animal Science, Shanxi Agricultural University, Taigu, China; ^5^ Department of Gastroenterology, Affiliated Children’s Hospital of Soochow University, Suzhou, China

**Keywords:** *Bifidobacterium longum*, *Pediococcus pentosaceus*, probiotic, infantile colic, prebiotics

## Abstract

The combination of *Bifidobacterium longum* and *Pediococcus pentosaceus* is a clinically effective probiotic formulation for alleviating infantile colic; however, their utilization characteristics and mechanism of action surrounding their combined use of sugar sources remains unclear. Using *in vitro* simulation technology, this study set up individual and mixed cultures of the two probiotics at unique concentrations, and different types of prebiotics, carbohydrates and polyols were added. Gas and short-chain fatty acid production, substrate utilization, as well as growth of the individual and mixed probiotics were detected at the beginning of fermentation, 24 h, and 48 h. Further, the mechanism of the syntrophic effect of the two probiotics was explored based on their growth characteristics. It was found that neither strain produced gas after 24 h and 48 h of cultivation, but could synergistically utilize fructo oligosaccharides (FOS) when mixed. There was an increasing trend of acetic acid production for *B. longum* in yeast extract, casitone and fatty acid (YCFA) and FOS medium with increasing of bacterial concentrations at 24 h and 48 h; whereas the trend for *P. pentosaceus* was less obvious. When bacterial concentrations were >5 billion CFU·g^−1^, the mixed culture showed significantly lower acetic acid production than *B. longum* alone. By adding lactic and acetic acids to the YCFA medium and observing *P. pentosaceus* growth, the results suggested that *Pediococcus pentosaceus* could use the acetic acid and lactic acid produced by *Bifidobacterium longum* for growth. When the bacterial concentration was 5 billion CFU·g^−1^, the acetic acid production of *B. longum* was significantly higher in the mixed cultures in lactulose, lactose, FOS, galactooligosaccharide, and inulin medium; whereas the reverse was true for culturing in xylitol, carboxymethyl cellulose sodium, and sorbitol medium. Further, the mixed cultures produced significantly more acetic acid than *B. longum* alone. In summary, through *in vitro* simulation experiments, the optimal ratio and potential interaction mechanisms between *B. longum* and *P. pentosaceus* were revealed here, offers a basis for understanding how the probiotic combinations may improve infant colic symptoms by influencing the gut pH and regulating the gut microbiota mechanisms.

## 1. Background

Infantile colic, categorized as a functional colic, is a behavioral syndrome characterized by long periods of crying and difficulty in soothing often occurring shortly after birth (1). Infantile colic was initially described by Wessel et al. (2) in the 1950s, and has since undergone several revisions (3, 4). The current clinical diagnostic criteria for infantile colic are that the infant is <5 months old when the symptoms occurred and stopped, without any obvious cause creating the prolonged phases of crying or irritability. In addition to meeting the diagnostic criteria, clinical research cases also require the presence of symptoms lasting >3 h for ≥3 days per week, or >3 h of crying and irritability within a 24 h period. The occurrence of infantile colic causes anxiety for parents, and also poses challenges for clinical practitioners. Currently, the pathogenesis of infantile colic has not yet been fully elucidated.

Several studies have shown that gut microbiota dysbiosis, gastrointestinal hormone changes, neurodevelopment, as well as psychosocial and other factors are all potential causes of infantile colic (5). Namely, gut microbiota has been a recent focus of scholars, and the results showing that the diversity of gut microbiota in infants with colic is varied, with an increased abundance of *Escherichia coli*, *Klebsiella*, and *Vibrio*, while the levels of *Bifidobacterium* and lactic acid bacteria are significantly decreased (6, 7). These findings supported the possibility for the treatment of infantile colic using probiotics. Namely, *Bifidobacterium longum* and *Pediococcus pentosaceus* are probiotics isolated from healthy children's fecal samples, and their combination has been marketed as a probiotic product. To date, numerous studies have evaluated their efficacy and safety. Chen et al. (8) conducted a 21-day double-blind randomized controlled trial involving 112 infants with colic. and found that daily oral administration of a certain dose of the two-strain probiotic combination effectively reduced the duration and frequency of crying in these infants. Furthermore, as the probiotic use continued, time and frequency of crying continued to decrease, in addition to significant improvements in the stool properties of infants. Erola et al. (9) similarly demonstrated in their research that the two-strain probiotic combination had a synergistic effect in increasing the diversity of intestinal adhesion proteins during the treatment of infantile colic, in addition to validating its safety and efficacy.

Given the significant efficacy of *B. longum* CECT7894 and *P. pentosaceus* CECT8330 in the treatment of infantile colic, we hypothesize potential interaction mechanisms between the two probiotic strains. Additionally, there may exist an optimal ratio between them to achieve the best therapeutic outcomes. Therefore, this study explored the *in vitro* biological characteristics and mechanisms of *B. longum* CECT7894 and *P. pentosaceus* CECT8330 using an *in vitro* fermentation simulation system. We aim to elucidate the potential mechanism of action in treating infant colic by examining the interaction between probiotics and their combined effects on the gut microbiota. This research seeks to provide a theoretical foundation for advancing the treatment approaches for infant colic in the future.

## 2. Methods

### 2.1. Bacterial strains and growth conditions

*B. longum* CECT7894 and *P. pentosaceus* CECT8330 were provided by DiPROBIO Co., Ltd (Shanghai, China). *P. pentosaceus* CECT8330 was routinely grown in de Man, Rogosa and Sharpe (MRS) medium broth for 18–48 h at 37 °C in a chamber with anaerobic pouches. Comparatively, *B. longum* CECT7894 was grown under similar conditions, with 0.1% (w/v) cysteine-HCl being added to the MRS broth. Bacterial suspensions of different concentrations of *B. longum*, *P. pentosaceus*, and their mixed cultures were prepared: 50 million, 100 million, 500 million, 1 billion, 5 billion, and 10 billion CFU·g^−1^.

The composition of the YCFA medium used for *in vitro* fermentation has been shown in Table 1.

**Table 1:** YCFA medium Formula.

YCFA medium formula	
YCFA	g/L
Tryptone	10
Yeast extract	2.5
L-cysteine	1.0
Hemin (ml)	2.0
NaCl	0.9
CaCl_2_·6H_2_O	0.09
KH_2_PO_4_	0.45
K_2_HPO_4_	0.45
MgSO_4_·7H_2_O	0.09
Vitamin I (μl)	200
1 mg/ml Resazurin (ml)	1.0
Soluble starch (g)	8.0

Vitamin I: VH 2 mg, Vb12 2 mg, 4—(Aminomethyl) benzoic acid 6 mg, Folinic acid 10 mg, Vb6 30 mg; Vitamin II: VB1 5 mg, VB2 5 mg.

Based on the YCFA medium formula, 8 g·L^−1^ of substances corresponding to energy sources was added to prepare lactulose (LAU), lactose (LAT), fructo-oligosaccharide (FOS), galacto-oligosaccharide (GOS), inulin (INU), xylitol (XYI), carboxymethylcellulose sodium (CMS), and sorbitol (SBI) medium.

#### 2.1.1. Gas production measurement

A barometer (HT-1895, Beijing Xieya Electronics Co., Ltd., China) was used to detect the total gas production in fermentation bottles across different doses. The initial pressure value at 0 h was analyzed, and the barometric pressure values were measured again at 24 h and 48 h after removing the bottles from the incubator.

### 2.2. Short-chain fatty acid detection

A total of 0.5 ml of the fermentation liquid was pipetted into a 1.5 ml microcentrifuge tube. Next, 0.1 ml of crotonic acid was added, and mixed well, before storing the mixture at −30 °C overnight. Short chain fatty acids (SCFAs) were detected and analyzed using gas chromatography (GC9720II; Zhejiang Fuli Analytical Instruments Inc., China) with the Agilent FFAP 30 m × 0.25 mm × 0.25 μm chromatographic column at 75 °C. The column temperature was increased at a rate of 20 °C min^−1^ to 180 °C. and held for 1 min, followed by raising the temperature at 50 °C min^−1^ to 220 °C. and held for 1 min. The temperature at the injection port was 220 °C, and the injection volume was 1.0 μl, with a split ratio of (5:1). A high-purity nitrogen carrier gas was used at a flow rate of 2.5 ml·min^−1^. An FID detector, with a detection temperature of 250 °C was used as well. Tail-blowing was 30 ml·min^−1^, with hydrogen at 30 ml·min^−1^, and air at 300 ml·min^−1^.

### 2.3. Substrate degradation rate detection

Thin-layer chromatography (TLC) was used to detect the substrate degradation rate. A 0.2 μl sample was evenly applied to the marked position on the chromatographic plate, and approximately 35 ml of developing solvent was maintained in the chromatographic tank. When the solvent front reached the top of the plate, the plate was quickly removed and dried in a fume hood. The dried plate was quickly immersed in the staining solution, and hot air was blown using a heat gun at the maximum setting until the color appeared before scanning. The scanned images were processed using the “TLC” software. The degradation rate was calculated according to Equation (1):

(1) $$\frac{\left(\begin{array}{c} \mathrm{mean}\;\mathrm{grayscale}\;\mathrm{value}\;\mathrm{of}\;\mathrm{sample}\;\mathrm{at}\;0\;\mathrm{hr}\\ -\;\mathrm{mean}\;\mathrm{grayscale}\;\mathrm{of}\;\mathrm{sample}\;\mathrm{at}\;24\;\mathrm{hr} \end{array}\right)}{\mathrm{mean}\;\mathrm{grayscale}\;\mathrm{value}\;\mathrm{at}\;0\;\mathrm{hr}}$$

### 2.4. Probiotic concentration measurement

A qPCR machine (Singu9600, Singuway Biotech Inc.; Shenzhen, China) was used to analyze the concentration of probiotics. Specific primers for *B. longum* (BiLON-1 TTCCAGTTGATCGCATGGTC/BiLON-2 GGGAAGCCGTATCTCTACGA) and *P. pentosaceus* (ldhDF-GGACTTGATAACGTACCCGC or ldhDRGTTCCGTCTTGCATTTGACC) were used to quantify the corresponding amounts in different culture medium through qPCR.

### 2.5. Strain identification

After culturing the strains, DNA was extracted and subjected to 16S full gene sequencing for verifying the accuracy of strain isolation. Following several rounds of isolation and purification, the purified strains of *B. longum* and *P. pentosaceus* were obtained through 16S sequencing.

### 2.6. Metabolite characteristics of *B. longum*, *P. pentosaceus*, and mixed strains

For each of the two individual strains and their mixed culture, 0.5 ml of a bacterial suspension at 6 different concentrations (50 million, 100 million, 500 million, 1 billion, 5 billion, and 10 billion CFU·g^−1^) was inoculated into YCFA and FOS medium. For each bacterial suspension and concentration, two parallel samples were set up at two time points (24 h and 48 h). The samples were incubated at 37 °C for 24 h and 48 h, while the gas pressure, SCFAs, and substrate degradation rates were measured at 24 and 48 h.

### 2.7. Metabolic characteristics of *B. longum*, *P. pentosaceus*, and mixed strains in different prebiotics

Bacterial suspensions of the two individual strains and their mixed culture at 3 different concentrations (50 million, 500 million, and 5 billion CFU·g^−1^) were added to the medium containing different prebiotic substrates. The SCFAs, substrate degradation rates, and growth of probiotics were measured at 24 and 48 h. Based on the YCFA medium formula, 8 g·L^−1^ of substances corresponding to energy sources was added to prepare lactulose (LAU), lactose (LAT), fructo-oligosaccharide (FOS), galacto-oligosaccharide (GOS), inulin (INU), xylitol (XYI), carboxymethylcellulose sodium (CMS), and sorbitol (SBI) medium.

### 2.8. Mechanisms of syntrophic effects between *B. longum* and *P. pentosaceus*

Different concentration gradients of *P. pentosaceus* were prepared. The optical density (OD) corresponding to the bacterial liquid concentration was measured at a wavelength of 600 nm using a spectrophotometer (WFJ7200, Unico; Shanghai, China), and a standard curve was established. Based on the YCFA medium, medium containing 0, 10 mM, 25 mM, and 50 mM acetic and lactic acids were prepared. *P. pentosaceus* was inoculated at a concentration of 50 million CFU·g^−1^ into the above medium, and incubated at 37 °C for 24 h. A spectrophotometer was then used to continuously detect the OD values, and bacterial growth was observed.

### 2.9. Statistical analysis

Statistical analysis was performed using GraphPad Prism Software 9.0 (GraphPad Software Inc., La Jolla, CA, United States). Data from this study were expressed as Mean ± SEM and statistical significance of the treatment effect from this study was analyzed using one-way analysis of variance (ANOVA).

## 3. Results

### 3.1. Metabolic characteristics of *B. longum*, *P. pentosaceus*, and mixed strains in YCFA and FOS medium

**Figure 1:**
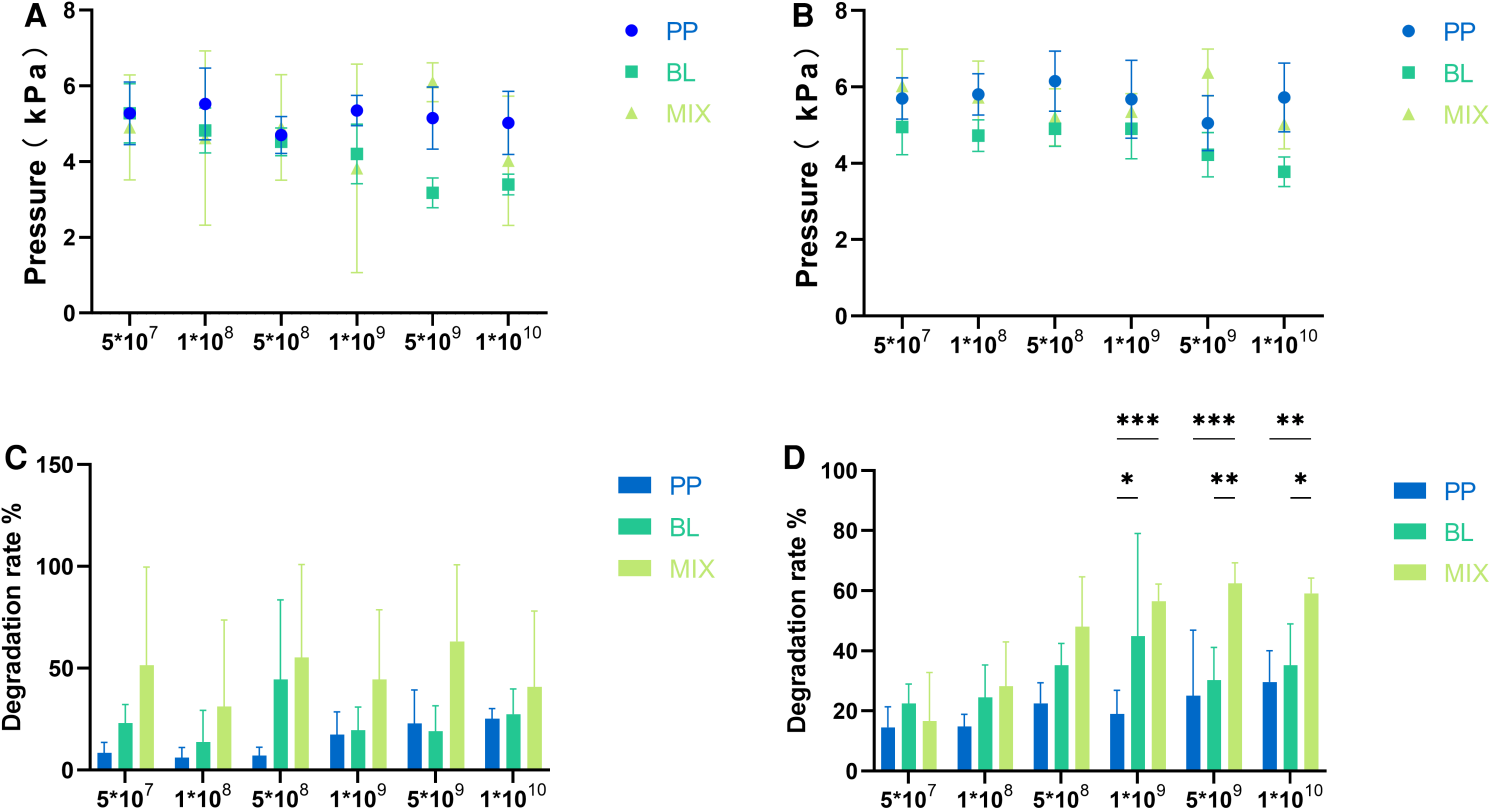
Gas production and degradation rates of individual and mixed strains. (**A**,**B**) Represent the gas production of *B. longum* (BL) and *P. pentosaceus* (PP), as well as their mixed culture (MIX) at 24 and 48 h, respectively. (**C**,**D**) Represent the differences in degradation rates among the three strains at 24 and 48 h, respectively. All data are expressed as means ± SEM (*n* = 3), and analyzed via one-way a ANOVA. Differences were considered significant at *p* < 0.05 (*), *p* < 0.01 (**), *p* < 0.001 (***), and *p* < 0.0001 (****).

There were no significant differences in the gas produced after fermentation at different concentrations for *B. longum*, *P. pentosaceus*, and the mixed strains (*p* > 0.05), indicating that both strains did not produce gas throughout the fermentation process (Figures 1A,B).

The degradation rates of FOS at 48 h were greater than those at 24 h for *B. longum*, *P. pentosaceus*, and the mixed strains across different concentrations. Further, the degradation rate increased with culture concentrations (Figures 1C,D).

**Figure 2:**
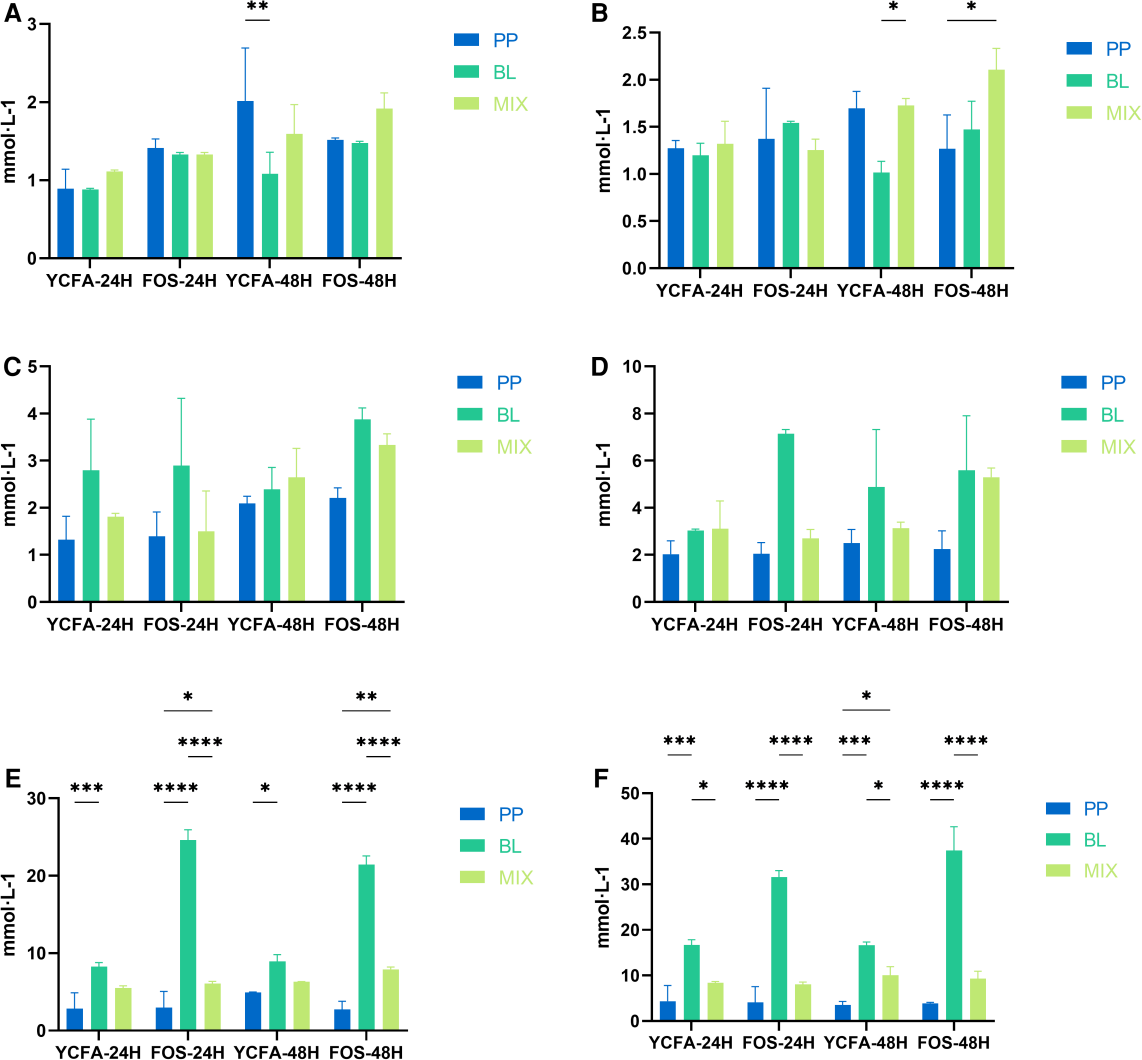
Acetic acid production at varying concentrations: (**A**–**F**) represent the acetic acid production of *B. longum* (BL), *P. pentosaceus* (PP), and their mixed culture (MIX) in YCFA and FOS medium at 50 million (**A**), 100 million (**B**), 500 million (**C**), 1 billion (**D**), 5 billion (**E**), and 10 billion (**F**) CFU·g^−1^ concentrations, respectively. All data are expressed as means ± SEM (*n* = 3), and analyzed via a one-way ANOVA. Differences were considered significant at *p* < 0.05 (*), *p* < 0.01 (**), *p* < 0.001 (***), and *p* < 0.0001 (****).

As shown in Figures 2A–F, with increasing cell concentrations and length of fermentation, the acetic acid production of *B. longum* showed an upward trend; whereas the trend for *P. pentosaceus* was not evident. Moreover, when the cell concentration was >500 million, the acetic acid production of *B. longum* at 24 and 48 h was significantly higher than that of *P. pentosaceus* (*p* < 0.05), indicating the latter had a poorer ability to produce acetic acid when FOS served as the carbon source. At strain concentrations of 50 million, 100 million, 500 million, and 1 billion, there were no significant differences in acetic acid production between *B. longum* and the mixed culture; however, when cell concentrations reached 5 billion and 10 billion, the acetic acid production of the mixed culture at 24 and 48 h was significantly lower than that of *B. longum* (*p* < 0.05), suggesting that when co-cultivated and in the presence of sufficient substrate, acetic acid may be metabolized through a certain pathway or YCFA composition is not appropriate for *B. longum*.

### 3.2. Metabolic characteristics of *B. longum*, *P. pentosaceus*, and mixed strains in different medium substrates

**Figure 3:**
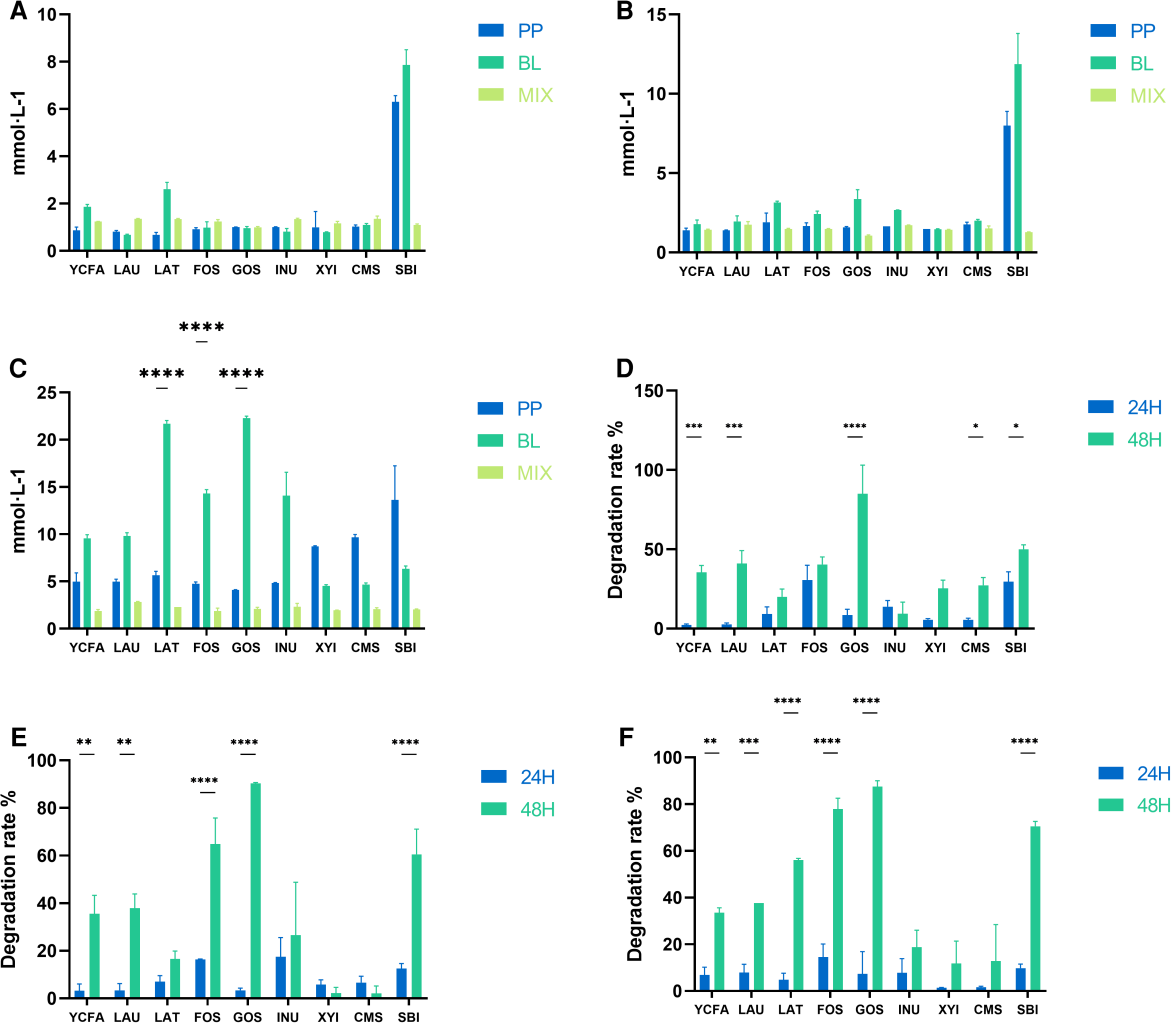
Acetic acid production and substrate degradation rates across various medium. (**A**–**C**) Represent the acetic acid production of *B. longum* (BL), *P. pentosaceus* (PP), and their mixed culture (MIX) in YCFA, LAU, LAT, FOS, GOS, INU, XYI, CMS, and SBI medium at 50 million (**A**), 500 million (**B**) and 5 billion (**C**) CFU·g^−1^, respectively. (**D**–**F**) Represent the comparison of substrate degradation rates for the mixed culture (MIX) 50 million (**D**), 500 million (**E**) and 5 billion (**F**) CFU·g^−1^. All data are expressed as means ± SEM (*n* = 3), and analyzed via one-way ANOVA. Differences were considered significant at *p* < 0.05 (*), *p* < 0.01 (**), *p* < 0.001 (***), and *p* < 0.0001 (****).

Both *B. longum* and *P. pentosaceus* produced more acetic acid in the SBI medium within 24 h at concentrations of 50 million and 500 million (Figures 3A–C); however, the production of acetic acid significantly decreased when the two strains were mixed. Save this lone exception, there was no significant differences in acetic acid production among the two strain combinations in other culture medium. When the bacterial concentration reached 5 billion, the acetic acid production of *B. longum* was significantly higher than that of the mixed culture in LAU, LAT, FOS, GOS, and INU medium; whereas in XYI, CMS, and SBI medium, these patterns were reversed, with the mixed culture produced significantly more acetic acid than *B. longum*.

When the mixed culture concentrations were 50 million, 500 million, and 5 billion, the degradation rates of prebiotics at 24 h and 48 h were significantly different, generally showing higher degradation rates at 48 h (Figures 3D–F). This indicated that with time, a synergistic acceleration of substrate utilization occurred between the two strains in different culture medium.

**Figure 4:**
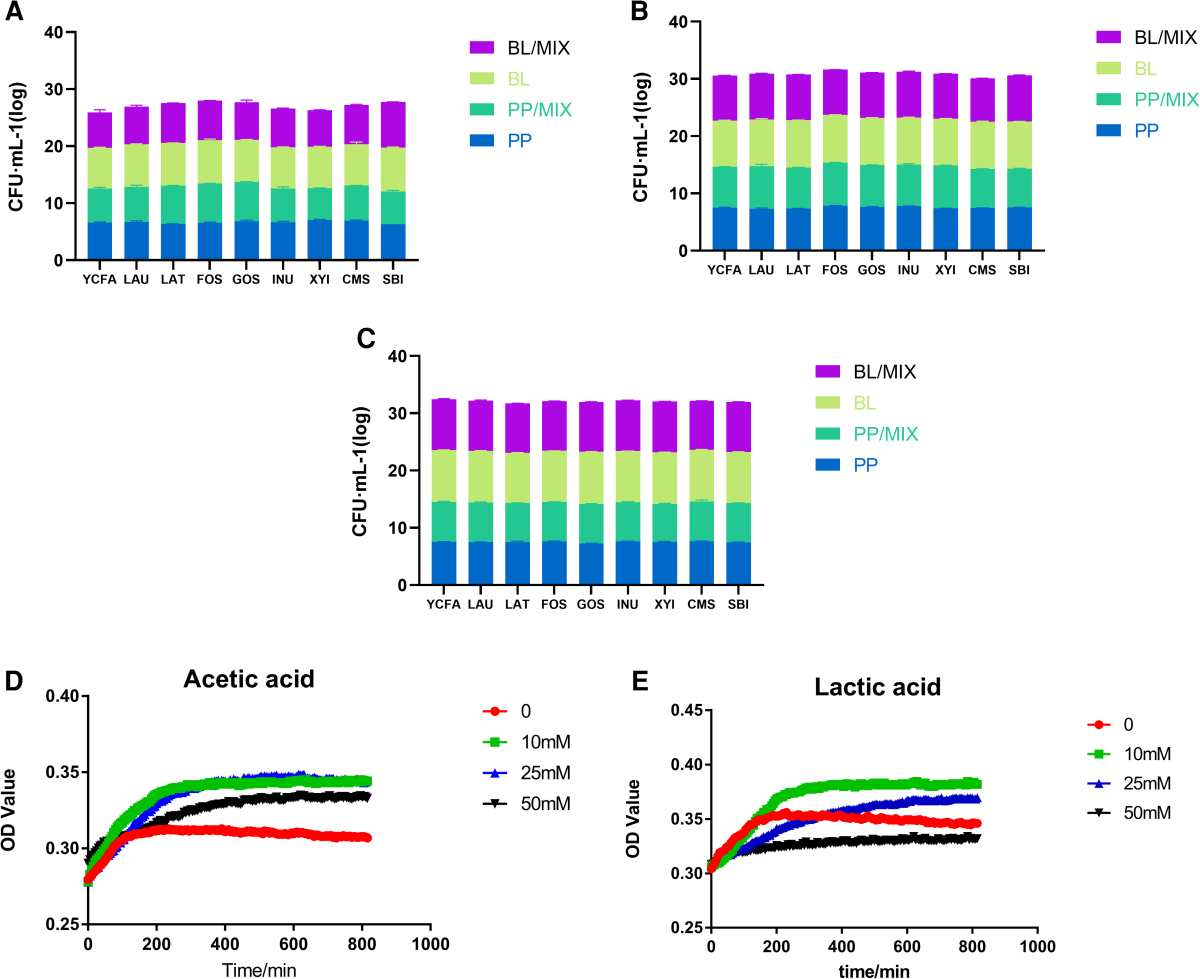
Growth of probiotics in various medium. (**A**–**C**) Represent the growth of *B. longum* (BL) and *P. pentosaceus* (PP) individually, and in mixed cultures, respectively, with YCFA, LAU, LAT, FOS, GOS, INU, XYI, CMS, and SBI medium over 48 h. (**D**,**E**) Represent the growth of *P. pentosaceus* at 0, 10, 25, and 50 mM concentrations of acetic and lactic acids, respectively.

The 48 h qPCR results showed that there were no significant differences in the growth-promoting effects of different prebiotics on *B. longum*, *P. pentosaceus*, and the mixed culture at concentrations of 50 million, 500 million, and 5 billion (Figures 4A–C).

### 3.3. Mechanism of syntrophic effects between *B. longum* and *P. pentosaceus*

Based on the above results, it was found that the acetic acid production of *B. longum* and within the mixed culture at bacterial concentrations of 5 billion in FOS, GOS, and LAT medium showed significant differences. There, the acetic acid production of *B. longum* was between 20 and 25 mM; therefore, different concentrations of acetic acid were used to explore its role on the growth of *P. pentosaceus*.

Under acetic acid concentrations of 0, 10, 25, and 50 mM, the growth of *P. pentosaceus* was significantly higher than that of the control group (0 mM; Figure 4D); however, compared to 10 and 25 mM concentrations, *P. pentosaceus* growth at 50 mM decreased, indicating that the optimal growth concentration of *P. pentosaceus* was around 25 mM acetic acid. Therefore, when acetic acid concentrations reached 50 mM, the survival rate of *P. pentosaceus* began to decline.

Under lactic acid concentrations of 0, 10, and 25 mM, the growth rate of *P. pentosaceus* was higher than that of the control group (0 mM), indicating that this acid also had a stimulating effect on the growth of *P. pentosaceus* (Figure 4E). Furthermore, the number of *P. pentosaceus* began to decrease when lactic acid concentrations reached 25 mM, and began to inhibited its growth at concentrations exceeding 50 mM.

## 4. Discussion

Previous studies have suggested that the supplementation of *Lactobacillus* and *Bifidobacterium* is due to the lower numbers of these two types of bacteria, and higher number of Gram-negative bacteria in the feces of infants with colic (10), indicative of the detrimental effects relating to the diversity of an infant's gut microbiota. Savino et al. (11) cultured and identified the feces of 45 infants with colic, revealing that the number of *Escherichia coli* in the culture medium with lactose as the carbon source was significantly higher than that in healthy infants, and all isolated strains, including *E. coli*, *Klebsiella pneumoniae*, *Enterobacter aerogenes*, and *Enterococcus faecalis,* could produce gasses. The authors subsequently confirmed *in vitro* that certain lactic acid bacteria could interact with gas-producing *E. coli*, thus inhibiting *E. coli* or gas production. Similarly, Aloisio et al. (12) also confirmed that certain *B. longum* and *Bifidobacterium breve* could play a role in the treatment of infants with colic by inhibiting the activity of gas-producing *E. coli*. *Bifidobacteria* can produce thiamine, riboflavin, vitamin B6, and vitamin K and have the capability to synthesize folate, niacin, and pantothenic acid. They metabolize carbohydrates primarily through the fructose 6-phosphate pathway, in which fructose 6-phosphate phosphoketolase serves as the key enzyme with dual-substrate specificity. The end metabolites of this metabolism are acetate, lactate, and ethanol (13). In the present study's *in vitro* fermentation model, there were no significant changes in gas content following fermentation of different concentrations of *P. pentosaceus*, *B. longum*, and the mixed strains in YCFA and FOS medium, this suggests that the consumption of a specific dose of the mixed strains may not lead to an increase in gas production within the human gut.

Although the combination of *B. longum* and *P. pentosaceus* has been clinically proven to be effective in the treatment of infantile colic, few studies have investigated the corresponding mechanisms of action in the intestine. Astó et al. (9) conducted *in vitro* experiments on the potential mechanism of *B. longum* and *P. pentosaceus* in the treatment of infantile colic, finding that both strains could tolerate gastric acid, bile salt, as well as other environmental conditions, and could effectively adhere to human intestinal epithelial cells, while synergistically regulating the expression of tight junction-related genes. Notably, these characteristics are crucial for maintaining the integrity of the intestinal barrier. Astó also found that both strains could produce bacteriocins *in vitro*, and it was speculated that this may be the potential combination mechanism of the two strains for inhibiting intestinal pathogens.

Previous studies have primarily focused on how single or mixed strains can alleviate infantile colic (14–16), without delving into the interactions between the two strains. The present experiments found that during *in vitro* fermentation, as the bacterial concentration increased along with fermentation time, the acetic acid production of *B. longum* showed an upward trend; whereas that for *P. pentosaceus* was less obvious. This suggests that *P. pentosaceus* itself does not produce acetic acid. Interestingly, at bacterial concentrations of 5 billion and 10 billion, the acetic acid production of the 24 and 48 h mixed culture was significantly lower than that of *B. longum* alone, indicating that there was a syntrophic effect of metabolites between the two strains. To examine whether this phenomenon was universal, additional experiments were conducted using different culture medium substrates. Similar phenomena were observed in LAU, LAT, GOS, and INU medium. Concurrently, using qPCR to detect the content of *B. longum*, it was found that although the acetic acid content decreased, its growth was not affected, further indicating that acetic acid produced by *B. longum* may be consumed by *P. pentosaceus*. To explore the optimal acetic acid concentration for stimulating *P. pentosaceus* growth, acetic acid gradients were set at 0, 10, 25, and 50 mM. Spectrophotometer detection revealed that the growth of *P. pentosaceus* across these different concentrations was significantly higher than that for the control group (0 mM); however, compared to 10 and 25 mM, the growth rate of *P. pentosaceus* decreased at 50 mM, indicating that an acetic acid concentration of ∼25 mM was optimal for *P. pentosaceus* growth. In the first experimental stage here, it was observed that significant differences in acetic acid production occurred when *B. longum* and the mixed culture concentrations were 5 billion under FOS, GOS, and LAT medium. There, the acetic acid production of *B. longum* approximately fell between 20 and 25 mM, indirectly suggesting that when the bacterial concentration was ≥5 billion, *B. longum* and *P. pentosaceus* can have a good syntrophic effect without competition affecting strain growth. Accordingly, it was speculated here that this may be the optimal dose for use in the mixed strains; however, most probiotic products currently available on the market contain bacterial concentrations ranging between 50 million and 100 million CFUs. We hypothesize that this may be due to the ability of both probiotic strains to maintain their functionality within the host even at lower bacterial concentrations, thereby facilitating the regulation of gut pH and the balance of the intestinal microbiota, which in turn could alleviate symptoms of infantile colic. Further exploration of the underlying mechanisms is warranted in future studies.

Research has shown that lactic acid-producing bacteria are the dominant type in the infant gut (17). Infant colic is associated with abnormal metabolism of lactic acid in the intestine. An accumulation of lactic acid and its metabolites may lead to gastrointestinal discomfort. Lactic acid is also one of the metabolic products of *Bifidobacterium*. Here, acid concentration gradients were established, and it was revealed that the growth rate of *P. pentosaceus* was higher than that of the control group (0 mM) at 10 and 25 mM lactic acid, indicating the acid's capacity to stimulate the growth of *P. pentosaceus*. Furthermore, when the lactic acid concentration reached 25 mM, the number of *P. pentosaceus* began to decrease; whereas concentrations >50 mM inhibited the growth of *P. pentosaceus*. Therefore, it appears that lactic and acetic acid metabolic products of *B. longum* served as growth substrates for *P. pentosaceus*, while the combination of *B. longum* and *P. pentosaceus* exhibited a syntrophic effect. Acetic acid and lactic acid can decrease the pH within the intestine. When *Pediococcus pentosaceus* utilizes acetic acid and lactic acid produced by *Bifidobacterium longum*, the acidity of the intestinal environment may change. This pH alteration aids in inhibiting the growth of specific acid-sensitive pathogens. This could be one of the potential mechanisms by which this combination effectively reduces excessive concentrations of acetic acid and lactic acid in the intestine to alleviate infant colic. In addition, we hypothesize that this syntrophic effect enables both types of probiotics to coexist and function effectively in the intestine. This maintains the balance of normal gut flora, ultimately alleviating symptoms of intestinal colic.

Dietary regimes containing varied prebiotics, carbohydrates, and polyols may have potential implications for intestinal contractile activity and the microbiota composition of infants with colic (18). In our study, we employed diverse growth medium to investigate the acetic acid production characteristics of *Bifidobacterium longum* and *Pediococcus pentosaceus*. Our findings revealed that at higher concentrations, distinct growth medium exhibited varied trends in acetic acid production. This underscores the significant influence of microbial concentration on the metabolic activity of bacterial strains across different medium. For instance, in the LAU, LAT, FOS, GOS, and INU medium, the acetic acid output of *Bifidobacterium longum* notably surpassed that of the mixed strains, suggesting a more efficient utilization of certain nutrients by *Bifidobacterium longum* in these medium, thereby enhancing its acetic acid metabolic pathway. Concurrently, we observed that in certain medium conditions, the acetic acid production by the mixed strains was actually lower than that by the individual strain, implying potential microbial competition or antagonistic interactions. Future research should be tailored to specific dietary habits, offering more precise growth conditions to further investigate the metabolic characteristics of this probiotic combination. For instance, studies could be designed to compare the effects of a high-fiber diet versus a high-sugar diet on the co-cultivation of these probiotics. Additionally, exploring how different dietary patterns, such as the Mediterranean diet, vegetarian diets, or a typical Western diet, affect the synergistic actions of these bacteria could be invaluable. Such research would contribute to a clearer understanding of how dietary habits influence the clinical efficacy of probiotics and provide a scientific basis for personalized probiotic therapies.

## 5. Conclusion

Using an *in vitro* fermentation simulation system, this study demonstrated the syntrophic effect between *B. longum* and *P. pentosaceus* in a commonly used clinical intervention combination and we hypothesize that this syntrophic effect may influence the gut microbiota by modulating the intestinal pH, ultimately leading to an improvement in the symptoms of infant colic. Furthermore, we conducted a series of gradient experiments to explore the optimal probiotic concentration that maximizes this syntrophic effect, providing a theoretical basis for future improvements to this product.

## Data Availability

The original contributions presented in the study are included in the article/Supplementary Material, further inquiries can be directed to the corresponding authors.
